# The incidence and prevalence of immunoglobulin A nephropathy in the United States 

**DOI:** 10.5414/CN111489

**Published:** 2024-11-19

**Authors:** Marc DeCongelio, Sarah N. Ali, Martina Furegato, Sandipan Bhattacharjee, Ancilla W. Fernandes

**Affiliations:** 1 Oracle Life Sciences, Austin, TX, USA,; 2 Otsuka Pharmaceutical Development and Commercialization, Inc., Princeton, NJ, USA,; 3 Oracle Life Sciences, Paris, France, and; 4 Otsuka Pharmaceutical Development and Commercialization, Inc., Princeton, NJ, USA (former)

**Keywords:** immunoglobulin A nephropathy (IgAN), epidemiology, electronic health records, biopsy, United States

## Abstract

Aims: Reliable national estimates for the incidence and prevalence of immunoglobulin A nephropathy (IgAN) in the United States (U.S.) are needed. We performed a national survey with pathologists and used insurance claims data to estimate IgAN frequency nationwide. Materials and methods: An online survey with pathologists was conducted between November and December 2021 to obtain data on the number and types of biopsies evaluated and the proportion with IgAN confirmed. Biopsy data were extrapolated to two different claims databases to estimate incidence and prevalence. Results were validated against a separate dataset of electronic health records. Results: A total of 43 pathologists from across U.S. regions reported evaluating a mean of 169 kidney biopsies (standard deviation 179.1) in the past 12 months. Of the 7,267 total biopsies evaluated, 632 (8.7%) were IgAN. Based on the respective claims databases, annual incidence rates of 2.1 and 2.2 per 100,000 and prevalence rates of 59.9 and 62.7 per 100,000 were estimated. Results from the validation dataset were similar, with an incidence of 1.9 per 100,000 and prevalence of 54.2 per 100,000. Conclusion: This study estimated incidence and prevalence of IgAN. Extrapolating the findings to the U.S. population for 2021, total prevalence was 198,887 – 208,184 persons.

## Introduction 

Immunoglobulin A nephropathy (IgAN) is one of the most common forms of primary glomerulonephritis (GN) worldwide [1]. Epidemiology varies by geography, with differences by region and data source attributed to variations in screening practices, cutoffs for laboratory values leading to nephrologist referrals, racial/ethnic population distribution, and frequency of kidney biopsies at different types of treatment center [1]. The lack of diagnostic codes specific for IgAN until recently (October 2023) in the International Statistical Classification of Diseases and Related Health Problems, Tenth Revision (ICD-10), has deterred the identification of confirmed diagnoses in claims data [2]. 

Incidence estimates worldwide range from 2 – 10 cases per 100,000, varying by region [3]. Prevalence estimates are higher for East Asia compared to Europe, while the lowest prevalence is observed in Africa [3, 4, 5]. Geographic variation in sex distribution also has been noted, with males affected more than females in North America and Europe but both sexes affected equally in East Asia [5]. While epidemiologic data is available from different global geographies, information related to the United States (U.S.) is limited to specific regions of the country [6, 7, 8, 9, 10, 11]. Thus, to develop a better understanding of the incidence and prevalence of IgAN in the U.S., a survey of biopsies with IgAN confirmed and total biopsy counts was performed. The proportion of kidney biopsies that were IgAN confirmed was then applied to nationwide samples of kidney biopsy medical claims and validated using electronic health record (EHR) data. 

## Materials and methods 

**Figure 1.**
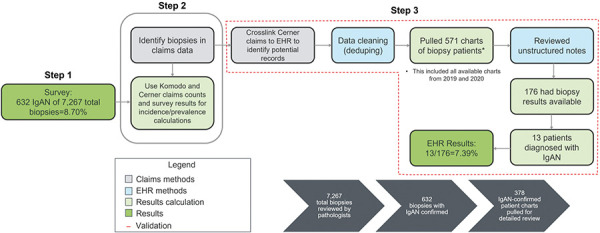
Study flow chart. EHR = electronic health record; IgAN = immunoglobulin A nephropathy.

The study design comprised three steps (Figure 1). In step 1, primary data were collected from board-certified pathologists who met the following inclusion criteria: medical specialty in pathology, ≥ 3 years in practice, examined ≥ 20 kidney biopsies in the past 12 months, examined ≥ 10 kidney biopsies with GN in the past 12 months, examined ≥ 1 kidney biopsy with GN that was classified as IgAN in the past 12 months, and uses mesangial deposition of immunoglobulin A on immunofluorescence to diagnose IgAN. 

Respondents completed a web-based questionnaire between November and December 2021, describing their evaluation of kidney biopsies in the last 12 months. The physicians estimated the number and types of biopsies evaluated and provided anonymized patient-level information including sex, race/ethnicity, and age from the charts of patients with biopsy-confirmed IgAN. These were limited to a convenience sample of the 20 most recent charts per pathologist to mitigate respondent burden, a number determined to be acceptable during pre-survey testing with physicians. To minimize the likelihood of repeatedly counting the same patients, the institutional affiliation of each respondent was captured. Review of the data indicated that no participant’s data needed to be removed due to double-reporting with another participant. 

In step 2, to estimate disease incidence, the numbers of biopsies confirming IgAN and total biopsies from the survey were extrapolated to two large U.S. claims databases, Komodo Health and Oracle Life Sciences. Komodo Health (https://www.komodohealth.com/) includes prescription and medical claims of over 120 million individuals from 150+ private insurers in the U.S., including Medicaid managed care and Medicare Advantage plans. Oracle Life Sciences has partnered with a major provider of closed claims data. This claims dataset integrates disparate sources of patient-level data to map the longitudinal patient journey. It employs de-identified, patient-level claims data, which contains prescription and/or medical claims of over 150 million individuals in the past 5 years, and over 90 million individuals in the past 12 months, collected from 150+ private insurers in the U.S., including Medicaid managed care and Medicare Advantage plans. For any given patient, while they are enrolled with a payer within the insurer sample, medical and/or pharmacy claims for that patient are available. 

The extrapolation in step 2 was based on the total number of kidney biopsy claims and the total numbers of individuals found in the claims data during the same timeframe as the biopsy survey. Annual incidence was estimated using the formula: 


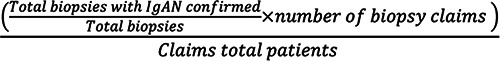


per 100,000 patients 

Prevalence per 100,000 population was estimated by multiplying incidence by disease duration determined from the published literature. 

To estimate disease duration, a life expectancy reduction in IgAN of 8 years was estimated by averaging the findings of Jarrick et al. [12] and Hastings et al. [13]. Given that life expectancy in the U.S. for 2021 as reported from the Centers for Disease Control and Prevention was 76.1 years, subtracting 8 years provided an estimate of life expectancy with IgAN of 68.1 years. The median age of IgAN onset based on our data, Hastings et al. [13], and Sim et al. [14] (i.e., 39.6 years) was then subtracted from the average life expectancy of 68.1 years in IgAN-affected individuals, yielding an estimated disease duration of 28.5 years. This calculation method assumes that the incidence rate is steady over a period (average incidence) indicated by duration of disease, with the assumption that the gradual accumulation of incident cases over time estimates current prevalence. Assuming stability over time, the rate is generalizable to the U.S. population. 

In the last step (step 3), the estimates obtained in step 2 were validated against an additional data source, Oracle Life Sciences EHR. Oracle Life Sciences EHR data is a national, de-identified EHR dataset which combines EHR data from all venues of care across 119 different health systems (including academic centers, Integrated Delivery Networks, and community hospitals) for over 100 million patients throughout the U.S.. Oracle Life Sciences EHR data is extracted from hospitals and clinics that have consented to such use. Encounters may include pharmacy, clinical and microbiology laboratory, admission, and billing information from affiliated patient care locations. All admissions, medication orders and dispensing, laboratory orders and specimens are date and time stamped, providing a temporal relationship between treatment patterns and clinical information. All data is de-identified in compliance with the Health Insurance Portability and Accountability Act. 

Oracle claims were linked to Oracle EHR data in step 3 to identify a subset of claims patients with available EHR data. EHR records were reviewed for biopsy results, and IgAN incidence was calculated. Prevalence was calculated by multiplying incidence by disease duration. EHR records were collated from 2019 and 2020. 

## Results 

The 43 pathologists who participated in the survey evaluated a mean of 169 (standard deviation (SD) 179.1) kidney biopsies in the past 12 months (Table 1). The mean duration in nephrology practice was 14.0 (SD 6.0) years. The region of practice was uniformly distributed across U.S. census regions: 25.6% in the Northeast, 25.6% Midwest, 25.6% South, and 23.3% West. Of the 7,267 total biopsies evaluated, 632 (8.7%) were IgAN. Among biopsies with GN classified (mean 64.1; SD 70.5), IgAN was the most common (mean 14.7; SD 26.2). 

Based on the respective claims databases, annual incidence rates per 100,000 were calculated: 

Komodo Health database 


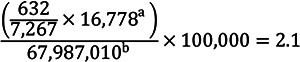


Oracle claims database 


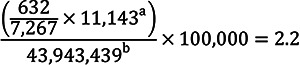


^a^Total number of kidney biopsy claims in the database for 2021.

^b^Total number of individuals in the claims data for 2021.

Multiplying incidence by disease duration of 28.5 years yielded prevalence rates of 59.9 per 100,000 for Komodo Health and 62.7 per 100,000 for Oracle claims. 

For the validation set, 571 patient charts with EHR data from 2019 – 2020 were extracted from the Oracle EHR and manually reviewed. The manual review was conducted by a siloed team of medically trained chart abstractors who had completed Health Insurance Portability and Accountability Act certification. Of the 571 patient charts, 176 had accessible biopsy results (some biopsy results were not accessible, as not all sites who contribute data to the database provide all data elements) detailed in physician notes, and 13 had a confirmed IgAN diagnosis. Extrapolating to the total number of Oracle kidney biopsy claims and total individuals in the claims data, incidence was 1.9 per 100,000 and prevalence was 54.2 per 100,000. 

Patient characteristics were provided from the chart review for a sample of 378 biopsy-confirmed individuals with IgAN, including age, sex, and race/ethnicity (Table 2). 

Based on prevalence estimates from the two main databases, the prevalence of IgAN extrapolated to the 2021 U.S. population of 332,031,554 [15] was: 

Komodo Health: (59.9/100,000) × 332,031,554 = 198,887 persons 

Oracle claims: (62.7/100,000) × 332,031,554 = 208,184 persons. 

## Discussion 

This study provides the incidence and prevalence of IgAN using large U.S.-based claims datasets and pathologist input. Prior local studies from the U.S. estimated the overall annual incidence of IgAN to be 2 – 3 per 100,000, which is consistent with our estimate of 2.1 – 2.2 per 100,000 [6, 7, 8]. An assessment of 35,967 native kidney biopsies obtained at 10 centers (n = 7 U.S., n = 3 Canada) by O’Shaughnessy et al. [9] found that 7.2% were IgAN, and a study of 4,128 native kidney biopsies collected at U.S. sites, primarily in Ohio and Florida, by Bobart et al. [10] indicated that 7.7% were IgAN. The 8.7% rate of biopsy-confirmed IgAN in our data is similar to the estimates from these multicenter North American studies [9, 10]. 

The age and sex data in our sample of biopsy-confirmed patients with IgAN are also consistent with reports of primary IgAN in the literature. The current cohort had a median age of 40 years (mean 41.1 (SD 17.3) years), similar to earlier studies (i.e., mean age 38.3 – 45.8 years) [9, 11, 16]. A greater proportion of patients in our study were males (63.0%) than females, consistent with studies by Selewski et al. [16] (61.8% pediatric, 58.9% adults), Sim et al. [11] (51.2% adults), O’Shaughnessy et al. [9] (62.1% all ages), and Bobart et al. [10] (62.7% all ages). 

Local U.S. studies by Swaminathan et al. [6] and Hommos et al. [8] were based in a Minnesota population that was largely White, and the population studied by Charu et al. [7] was from northern California, thus limiting generalizability to different racial/ethnic groups and geographical regions. The Cure GN Study, which is being conducted internationally but primarily in centers across the U.S., reported 77.0% of adult and 82.4% of pediatric IgAN patients as White, and 18.1% of adult and 12.2% of pediatric IgAN patients as Latino/Hispanic; sex and racial distribution was similar between adult and pediatric patients (p-value for comparison for sex, p = 0.51; race: p = 0.17) [16]. In the investigation of adult IgAN patients by Sim et al. [11], the proportions of Latino/Hispanic (38.6%), and Asian/Pacific Islander (30.4%) patients were higher compared to White (25.6%) and Black (3.1%) patients, which was representative of the demographics of southern California where the cohort was based. A high proportion of racial/ethnic data was missing from the study by O’Shaughnessy et al. [9] (i.e., for 46.1% of IgAN diagnoses), and Latinos/Hispanics and Asians were underrepresented in the population studied by Bobart et al. [10]. Given the limitations of the previous studies, it is difficult to compare the racial/ethnic results of our survey with data from existing literature. Future analysis of national samples is needed to understand if our findings are truly representative of the U.S. IgAN population. 

A limitation of our study is the restricted availability of biopsy results due to data agreements with Oracle. Also, the numerator from the claims data was the number of biopsy claims, not biopsy patients, with the consequent possibility that repeated biopsies in individual patients were captured. Additionally, EHR records with accessible data were collated from 2019 – 2020, while the claims and survey-based data were from a single year (2021). Data missingness is another issue for a retrospective analysis of patient charts, claims data, and EHR records. For example, of the 571 patient charts reviewed for biopsy information in the EHR validation set, only 176 charts had biopsy results. Pathologists provided in-depth information on a convenience sample of up to 20 patient charts, but many provided fewer than 20, which limits extrapolation to calculate age/sex/race-adjusted incidence/prevalence. Diagnostic codes specific for IgAN were not available in the ICD-10 until October 2023, after the period of study, further limiting the identification of confirmed diagnoses [2]. Future studies should aim to provide these missing data elements to complete the delineation of epidemiology of IgAN for the U.S.. A limitation of commercial claims data is that only patients with insurance are included, and therefore incidence and prevalence data for the uninsured population could not be estimated. Finally, the frequency of IgAN obtained from biopsy data was extrapolated to the claims database populations to derive incidence rates, instead of being calculated directly from population data. With the recent availability of ICD-10 codes for IgAN, future claims analysis studies could help confirm IgAN epidemiology estimates. 

In conclusion, the current study estimates IgAN incidence of 2.1 and 2.2 per 100,000 and provides valuable epidemiologic data on IgAN in the U.S. that is consistent with earlier studies. Extrapolating prevalence from the current analysis to estimate the total prevalence for the U.S. provides a range of 198,887 to 208,184 persons for 2021. 

## Acknowledgment 

The authors would like to thank Emeritus Professor Cynthia Willey, PhD, of the University of Rhode Island (Kingston, RI, USA), for sharing her expertise in the epidemiology of immunoglobulin A nephropathy and providing critical review of the manuscript. Editorial and writing services for this manuscript, funded by Otsuka, were performed by BioScience Communications, Inc. (New York, NY, USA). 

## Data sharing statement 

To submit inquiries related to Otsuka clinical research, or to request access to individual participant data (IPD) associated with any Otsuka clinical trial, please visit https://clinical-trials.otsuka.com/. For all approved IPD access requests, Otsuka will share anonymized IPD on a remotely accessible data sharing platform. 

## Authors’ contributions 

Conceptualization: M. DeCongelio, M. Furegato, S. Bhattacharjee. Methodology: M. DeCongelio, M. Furegato, S. Bhattacharjee, A.W. Fernandes. Data collection: M. DeCongelio, M. Furegato. Formal analysis: M. DeCongelio, M. Furegato, S.N. Ali. Interpretation: S.N. Ali. Supervision: A.W. Fernandes. Writing – original draft: S.N. Ali. Writing – review and editing: M. DeCongelio, S.N. Ali, M. Furegato, S. Bhattacharjee, A.W. Fernandes. 

## Funding 

This work was funded by Otsuka Pharmaceutical Development and Commercialization, Inc., Princeton, NJ, USA. The sponsor was involved in the study design; analysis and interpretation of data; in the writing of the report; and in the decision to submit the article for publication. 

## Conflict of interest 

S.N. Ali and A.W. Fernandes are employed by Otsuka Pharmaceutical Development and Commercialization, Inc., Princeton, NJ, USA, and S. Bhattacharjee was an Otsuka employee during the time the study was conducted. M. DeCongelio and M. Furegato are employed by Oracle Life Sciences, Austin, TX, USA, and Paris, France, respectively, which was retained by Otsuka to perform data collection and study analyses. 

**Table 1.** Pathologist (n = 43) characteristics and biopsies performed.

Question	
Primary medical specialty of pathology, n (%)	43 (100.0)
Board certified, n (%)	43 (100.0)
Number of years practicing in primary specialty, mean (SD)	14.0 (6.0)
Number of kidney biopsies examined in past 12 months, mean (SD)	169.0 (179.1)
Number of kidney biopsies examined in past 12 months with GN, mean (SD)	64.1 (70.5)
**Number of kidney biopsies examined in past 12 months with GN classified as (mean (SD)):**	
**IgAN**	**14.7 (26.2)**
Lupus nephritis	13.0 (23.8)
Infection-related GN	8.7 (13.8)
Fibrillary GN	3.7 (3.7)
ANCA-associated GN	7.5 (12.5)
Anti-glomerular basement membrane GN	5.0 (6.4)
Monoclonal Ig-GN	6.1 (7.8)
C3 GN	5.3 (6.6)
Of the IgAN biopsies, number which used MEST or MEST-C score to predict prognosis	12.5 (25.0)

ANCA = anti-neutrophilic cytoplasmic autoantibody; GN = glomerulonephritis; Ig = immunoglobulin; IgAN = immunoglobulin A nephropathy; SD = standard deviation.

**Table 2.** Patient chart demographics for biopsy-confirmed individuals with IgAN (n = 378).

Question	
Race/ethnicity^a^, n (%)	
African American/Black	73 (19.3)
Asian	59 (15.6)
American Indian	8 (2.1)
Latino/Hispanic	47 (12.4)
White	159 (42.1)
Other	3 (0.8)
Unknown	37 (9.8)
Age	
Mean (SD)	41.1 (17.3)
Median (min, max)	40 (4.0, 82.0)
Age category, n (%)	
< 18 years	28 (7.4)
18 – 24 years	47 (12.4)
25 – 34 years	65 (17.2)
35 – 44 years	81 (21.4)
45 – 54 years	60 (15.9)
≥ 55 years	97 (25.7)
Sex, n (%)	
Male	238 (63.0)
Female	136 (36.0)
Other (transgender, nonbinary, etc.)	4 (1.1)
Region of residence, n (%)	
Northeast	124 (32.8)
Midwest	78 (20.6)
South	105 (27.8)
West	71 (18.8)
Initial biopsy or follow-up, n (%)	
Initial biopsy	341 (90.2)
Follow-up biopsy	37 (9.8)
Tools used in diagnosis^a^, n (%)	
Mesangial deposition of IgA on immunofluorescence	378 (100.0)
Light microscopy	332 (87.8)
Electron microscopy	226 (59.8)
Other	3 (0.8)
Was the MEST or MEST-C score used to predict IgAN prognosis, n (%)	
Yes	290 (76.7)
No	88 (23.3)

^a^All categories that applied could be selected. IgA = immunoglobulin A; IgAN = immunoglobulin A nephropathy; max = maximum; min = minimum; SD = standard deviation.
